# Exploring anaerobic environments for cyanide and cyano-derivatives microbial degradation

**DOI:** 10.1007/s00253-017-8678-6

**Published:** 2017-12-05

**Authors:** Víctor M. Luque-Almagro, Purificación Cabello, Lara P. Sáez, Alfonso Olaya-Abril, Conrado Moreno-Vivián, María Dolores Roldán

**Affiliations:** 1 Departamento de Bioquímica y Biología Molecular, Universidad de Córdoba, Edificio Severo Ochoa, 1ª planta, Campus de Rabanales, 14071 Córdoba, Spain; 2 Departamento de Botánica, Ecología y Fisiología Vegetal, Universidad de Córdoba, Edificio Celestino Mutis, Campus de Rabanales, 14071 Córdoba, Spain

**Keywords:** Anaerobiosis, Biodegradation, Bioreactor, Cyanide, Cyanide-containing wastewaters, Metagenomics, Methanogenesis, Nitrilase, Nitrogenase, Thiocyanate

## Abstract

Cyanide is one of the most toxic chemicals for living organisms described so far. Its toxicity is mainly based on the high affinity that cyanide presents toward metals, provoking inhibition of essential metalloenzymes. Cyanide and its cyano-derivatives are produced in a large scale by many industrial activities related to recovering of precious metals in mining and jewelry, coke production, steel hardening, synthesis of organic chemicals, and food processing industries. As consequence, cyanide-containing wastes are accumulated in the environment becoming a risk to human health and ecosystems. Cyanide and related compounds, like nitriles and thiocyanate, are degraded aerobically by numerous bacteria, and therefore, biodegradation has been offered as a clean and cheap strategy to deal with these industrial wastes. Anaerobic biological treatments are often preferred options for wastewater biodegradation. However, at present very little is known about anaerobic degradation of these hazardous compounds. This review is focused on microbial degradation of cyanide and related compounds under anaerobiosis, exploring their potential application in bioremediation of industrial cyanide-containing wastes.

## Introduction: Cyanide in the environment. Forms, toxicity, sources and remediation

Chemical compounds that contain the cyano group (−C≡N) are usually called “cyanides.” In the environment, these compounds may be found in different forms including volatile hydrogen cyanide (HCN), simple inorganic salts (NaCN, KCN), metal-cyanide complexes with different stability and chemical composition, cyanate (OCN^−^), thiocyanate (SCN^−^), and organic cyanides (nitriles and cyanohydrins). Free forms of cyanide (HCN and CN^−^) are extremely toxic compounds; cyanate, thiocyanate, and nitriles are less toxic forms; and toxicity of metal-cyanide complexes depends on their capacity to break down releasing free cyanide (Baxter and Cummings 2006; Kumar et al. 2016). Cyanide acts as a potent metabolic poison because it tightly binds to metals, provoking the inactivation of metalloenzymes. In aerobic organisms, cyanide inhibits the cytochrome *c* oxidase, blocking the respiratory electron transport chain, and in animals, cyanide also reacts with methemoglobin in the bloodstream (Solomonson 1981; Jaszczak et al. 2017). Anaerobic microorganisms, especially methanogens, are even more sensitive to cyanide because they contain many relevant metalloproteins that are also inhibited in the presence of this toxic compound (Smith et al. 1985; Gijzen et al. 2000). Thus, cyanide toxicity threshold may be as low as 2 ppm for some anaerobes whereas is about 200 ppm for most aerobic microorganisms (Kuyucak and Akcil 2013).

Cyanide is usually found as pollutant in wastewaters from mining, jewelry, steel and metal industrial activities, production of chemicals, and food processing, among other processes. In addition, these industrial residues often contain important cyano-derivatives like cyanate, which results from cyanide oxidation, and thiocyanate, which is formed by the reaction between cyanide and reduced sulfur species (Akcil 2003; Luque-Almagro et al. 2016). Nitriles (R−C≡N) are also produced in the manufacture of feedstock, solvents, pharmaceuticals, and organic chemicals (Banerjee et al. 2002). Accumulation of cyanide-containing wastewaters in the environment becomes a potential risk to ecosystems and human health. Therefore, these industrial residues need to be treated by physical and/or chemical methods before discharging the effluents into the environment. These physical-chemical treatments operate predominantly under aerobic conditions, and they are expensive, require complex infrastructures, need hazardous reagents or generate toxic by-products, and are usually ineffective for stable metal-cyanide complexes (Akcil, 2003; Dash et al. 2009; Novak et al. 2013; Park et al. 2017).

Despite its toxicity, cyanide is a natural compound synthesized by a variety of organisms, including bacteria, fungi, plants, and animals, in which cyanogenesis may serve as defensive or offensive mechanism (Luque-Almagro et al. 2016). The HCN synthase required for bacterial cyanogenesis is expressed during transition from exponential to stationary phase of growth under oxygen limitation in response to the FNR-like anaerobic regulator ANR (Laville et al. 1998). On the other hand, many microorganisms have evolved enzymatic pathways for cyanide degradation, transformation, or tolerance, and many of them are even able to use cyanide as a nitrogen source for growth. Therefore, cyanide biodegradation has become an efficient economically interesting alternative to the physical-chemical treatments of cyanide-containing industrial residues (Ebbs 2004; Baxter and Cummings 2006; Dash et al. 2009; Kumar et al. 2016; Luque-Almagro et al. 2016; Park et al. 2017).

**Fig. 1:**
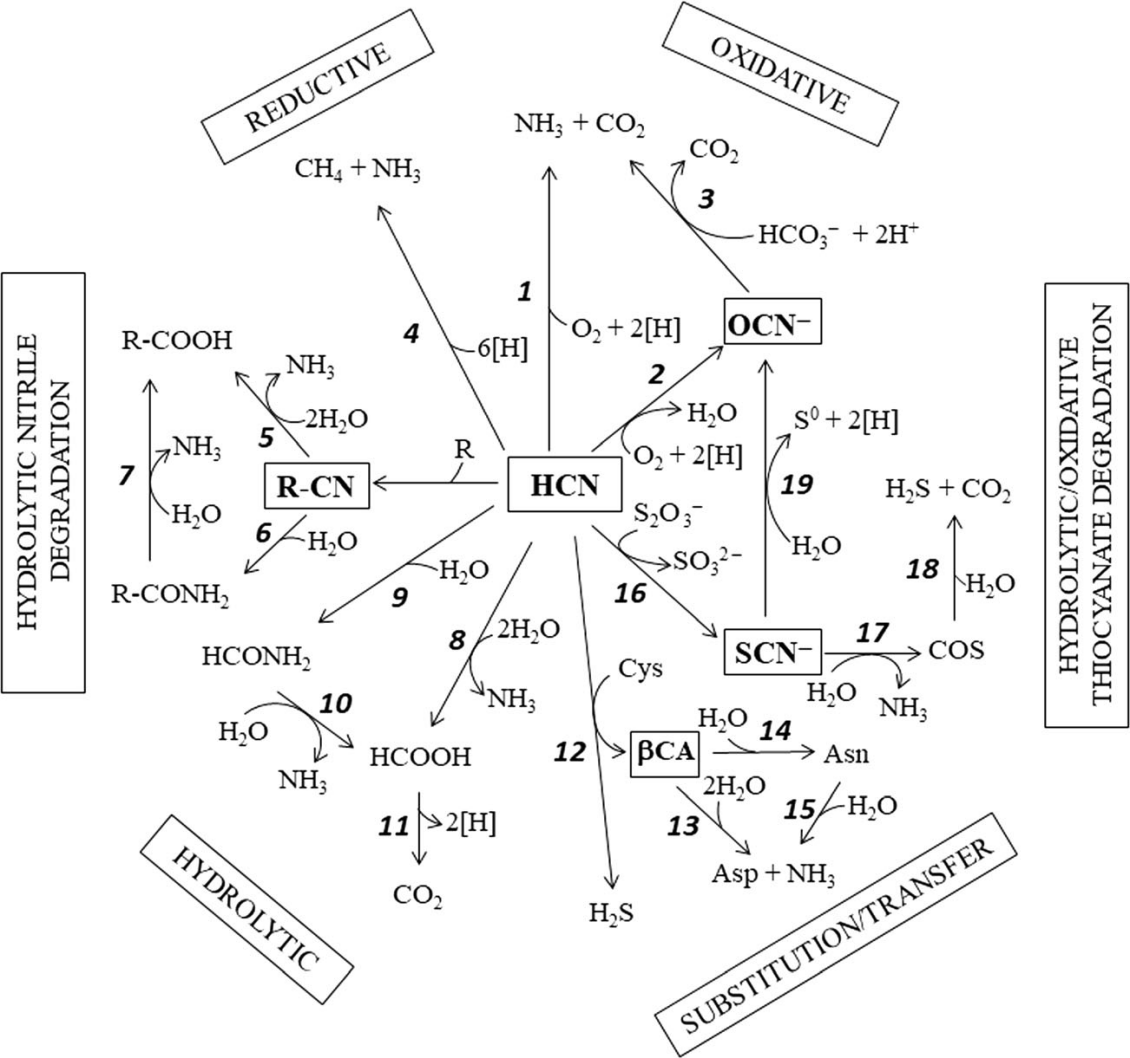
Biochemical pathways for the biodegradation of cyanide and its derivatives. The cyano-compounds are boxed and highlighted in bold. Symbols: R-CN, nitrile (organic cyanide); R, organic compound; [H], hydrogen atom (e^−^ + H^+^); βCA, β-cyanoalanine. Enzymes: 1, cyanide dioxygenase; 2, cyanide monooxygenase; 3, cyanase; 4, nitrogenase; 5, nitrilase; 6, nitrile hydratase; 7, amidase; 8, cyanidase (cyanide dihydratase); 9, cyanide hydratase; 10, formamidase; 11, formate dehydrogenase; 12, β-cyanoalanine synthase; 13, β-cyanoalanine nitrilase; 14, β-cyanoalanine hydratase; 15, asparaginase; 16, rhodanese; 17, thiocyanate hydrolase; 18, carbonyl sulfide (COS) hydrolase; 19, thiocyanate dehydrogenase

Microorganisms utilize different metabolic pathways to degrade or to assimilate cyanide. In general, these degradative routes are based in four types of enzymatic processes: hydrolytic, oxidative, reductive, and substitution/transfer reactions (Ebbs 2004; Huertas et al. 2006; Dash et al. 2009; Gupta et al. 2010; Park et al. 2017). These enzymatic degradation pathways are summarized in Fig. 1. The hydrolytic reactions are catalyzed by two different enzymes: the cyanidase (cyanide dihydratase) that transforms cyanide into formic acid and ammonia (Fig. 1; reaction 8), or the cyanide hydratase that produces formamide, which is further hydrolyzed by a formamidase (Fig. 1; reactions 9 and 10) (Martínková et al. 2015). The oxidative reactions generate carbon dioxide and ammonia either directly by the cyanide dioxygenase (Fig. 1; reaction 1) or in two-step reactions, via cyanate, catalyzed by the cyanide monooxygenase and the cyanase, respectively (Fig. 1; reactions 2 and 3) (Raybuck 1992; Ebbs 2004). The reductive pathway involves the nitrogenase required for biological nitrogen fixation, an oxygen-sensitive enzyme that also utilizes various substrates containing carbon-nitrogen triple bonds, such as hydrogen cyanide, nitriles, and isonitriles. Both molybdenum- and vanadium-nitrogenases carry out the six electrons reaction that converts HCN into methane and ammonia (Fig. 1; reaction 4) (Fisher et al. 2006; Seefeldt et al. 2013). Cyanide is also metabolized by the 3-cyanoalanine synthase (Fig. 1; reaction 12), which uses cysteine or *O*-acetylserine as substrate. The 3-cyanoalanine formed in this reaction can be further hydrolyzed to ammonia and aspartate in one-step reaction or with asparagine as intermediate (Fig. 1; reactions 13–15) (Howden et al. 2009). Finally, the rhodanese (thiosulfate:cyanide sulfurtransferase) catalyzes the reaction between cyanide and thiosulfate to form thiocyanate and sulfite (Fig. 1; reaction 16) (Cipollone et al. 2007). Another sulfurtransferase family enzyme, the 3-mercaptopyruvate sulfurtransferase, also transforms cyanide into thiocyanate but coupled to the conversion of mercaptopyruvate into pyruvate (Park et al. 2017).

Thiocyanate, a compound much less toxic than cyanate, can be used by different bacteria as a source of energy, carbon, sulfur, or nitrogen (Sorokin et al. 2001). Thiocyanate is degraded to ammonia, carbon dioxide, and sulfide, which may be oxidized to sulfate by chemolithotrophic sulfur-oxidizing bacteria. Two different hydrolytic degradative pathways, involving either carbonyl sulfide (COS) or cyanate as intermediates, have been proposed (Kelly and Baker 1990; Sorokin et al. 2014). In the COS pathway, the initial hydrolytic cleavage of the C≡N bond by the thiocyanate hydrolase generates ammonia and carbonyl sulfide as first products (Fig. 1, reaction 17). COS is further hydrolyzed to carbon dioxide and sulfide (Fig. 1; reaction 18), which is finally oxidized to sulfate. In the cyanate pathway, it was initially proposed that an uncharacterized enzyme hydrolyzes the C−S bond converting thiocyanate into cyanate and sulfide. Then, cyanate can be hydrolyzed to ammonia and carbon dioxide by the cyanase, whereas sulfide can be oxidized to sulfate. However, very recently it has been described that the initial step of the thiocyanate degradation pathway via cyanate is an oxidation reaction catalyzed by the thiocyanate dehydrogenase (thiocyanate:cytochrome *c* oxidoreductase), a copper-containing enzyme that converts thiocyanate into cyanate and elemental sulfur with cytochrome *c* acting as electron acceptor (Fig. 1; reaction 19) (Berben et al. 2017).

Microbial degradation of nitriles usually requires hydrolytic reactions that generate ammonia and the corresponding carboxylic acid. Nitrilases catalyze this conversion in a single reaction (Fig. 1; reaction 5), while nitrile hydratases generate an amide intermediate that is further hydrolyzed by an amidase (Fig. 1; reactions 6 and 7) (Kobayashi and Shimizu 1998, 2000; Park et al. 2017).

## Anaerobic degradation of cyanide

Cyanide is usually biodegraded through aerobic routes, according to the different pathways described above. However, microorganisms are also able to degrade cyanide under anaerobic conditions, although much more slowly and less successfully. The first description of an anaerobic cyanide biodegradation process was reported by Fedorak and Hrudey (1989) in methanogenic semicontinuous batch cultures. Since then, various applications based on anaerobic reactors or combining both aerobic and anaerobic processes have been developed for the treatment of different cyanide-containing wastewaters (Gijzen et al. 2000; Akcil and Mudder 2003; Chakraborti and Veeramani 2006; Novak et al. 2013; Joshi et al. 2016), although in general the anaerobic cyanide degradation process is not well understood and there is little knowledge about the microbial communities involved. In addition, abiotic anaerobic cyanide degradation may also occur when cyanide spontaneously hydrolyzes generating formic acid. In fact, a combination of simultaneous biotic and abiotic processes seems to contribute to the successful removal of cyanide in an upflow anaerobic sludge blanket (UASB) reactor (Novak et al. 2013).

Anaerobic biological treatments of wastewaters are attractive technologies that allow production of biogas with reduced biological oxygen demand (BOD) and low sludge volume and energy requirements, thus resulting in more cost-effective and energy-saving systems than aerobic procedures. In addition, anaerobic environments are not uncommon in nature, and in fact anaerobiosis prevails in most wastewater and polluted groundwater. Therefore, the identification of microorganisms able to produce methane in the presence of cyanide and the better understanding of the mechanisms involved in the anaerobic treatments of cyanide may convert this process into a feasible and efficient removal technology.

The chemical nature of the cyanide biodegradation reactions accounts for the fact that only the reductive or hydrolytic pathways may operate under anaerobic conditions (Fallon 1992). The reductive conversion of cyanide into methane and ammonia catalyzed by the nitrogenase has been described in resting cells of *Klebsiella oxytoca*, but not in cell-free extracts, probably due to the inactivation of the enzyme by oxygen exposure during cell disruption (Kao et al. 2003). Application of alginate and cellulose triacetate immobilized cells of *K. oxytoca* for the treatment of a cyanide-containing wastewater resulted in a more effective cyanide degradation, with higher tolerance to cyanide at wider ranges of pH, than when using free cells (Chen et al. 2008). This bacterium was also able to degrade the metal-cyano complex tetracyanonickelate (II) under anaerobic conditions, and nitrogenase was proposed to be the sole enzyme involved in this degradative process (Kao et al. 2004; Chen et al. 2009). However, the amount of cyanide removed by the nitrogenase in the environment is believed to be relatively small because this enzyme is rarely found in microbial populations (Gupta et al. 2010). On the other hand, it has been also demonstrated that hydrolytic reactions were responsible for anaerobic cyanide degradation in an upflow anaerobic fixed-bed reactor with activated carbon, transforming cyanide into ammonia and formic acid, which subsequently generated bicarbonate (Fallon et al. 1991; Fallon 1992). However, it was not possible to distinguish whether cyanide hydrolyzed directly or through the formation of formamide as an intermediate (Fallon 1992). Therefore, cyanide transformation analogous to hydrolytic reactions described for aerobes also occur in anaerobic systems. These hydrolytic pathways are probably the most attractive for biotechnological applications.

Nitrilases carry out the hydrolysis of the nitrile group to produce the corresponding carboxylic acid. Bacterial nitrilases show activities toward a wide range of nitriles, and are also able to degrade cyanide into ammonia and formate (Park et al. 2017). In addition, cyanide reacts chemically with different oxoacids to form cyanohydrins (hydroxynitriles), which may be hydrolyzed to ammonia and a carboxylic acid by a nitrilase enzyme, as described for the cyanide-degrading bacterium *Pseudomonas pseudoalcaligenes* CECT5344 (Estepa et al. 2012). Thus, nitrilases could be also good candidates for both aerobic and anaerobic cyanide remediation.

## Cyanide biodegradation and methanogenesis

Methanogenesis can be maintained under a variety of feed medium conditions, which include ethanol, methanol, phenol, and toxic compounds as the primary reduced carbon sources. Many industrial wastewaters contain cyanide and related compounds, but cyanide has been usually considered highly toxic for anaerobes, especially for methanogens, resulting in minimal attention to the anaerobic treatments.

In an UASB reactor using a synthetic wastewater containing starch and fatty acids, sludge was successfully acclimatized to high cyanide concentrations (up to 125 mg/L), allowing an elevated methane production with high cyanide degradation efficiency. Cyanide inhibition on methanogenic activity was more pronounced for acetoclastic than for hydrogenotrophic methanogens, suggesting that enzymes and cofactors involved in hydrogenotrophic methane production are less sensitive to cyanide (Gijzen et al. 2000). Acclimatization of anaerobic microbes to cyanide was also used to improve the degradation rates in an anaerobic batch reactor with sludge from a wastewater treatment plant and fresh cow dung (Gupta et al. 2016). In this study, it was also observed that hydrogen-utilizing methanogens were more tolerant to cyanide than acetate-utilizing methanogens (Gupta et al. 2016). Successful biogas production and cyanide removal without methanogenesis inhibition was also reported in an UASB reactor for brewery wastewater treatment (Novak et al. 2013). Anaerobic cyanide degradation resulted from a combination of both biotic and abiotic processes, and again, the hydrogenotrophic community was less sensitive to cyanide than the acetoclastic methanogens. The phylogenetic analyses carried out by 16S rRNA sequences allowed the identification of the bacterial phylum *Firmicutes* and the archaeal genus *Methanosarcina* as relevant microbial groups involved in the anaerobic cyanide degradation associated to methane production (Novak et al. 2013).

During the production of cassava starch, large amounts of cyanide are released from cyanoglycosides by hydrolytic enzymes present in the raw cassava peel, leading to a cyanide concentration in the wastewater as high as 200 mg/L. Thus, linamarase hydrolyzes the cyanoglycoside linamarin releasing cyanide, which is detoxified to 3-cyanoalanine by the 3-cyanoalanine synthase (Cuzin and Labat 1992). When a cassava root wastewater was fermented in an anaerobic fixed-bed methanogenic reactor to produce biogas, up to 150 mg/L cyanide could be removed after biofilm establishment. All nitrogen derived from cyanide was converted into organic nitrogen by the biomass (Siller and Winter 1998a). Anaerobic degradation of this cyanide-rich agroindustrial wastewater was optimized in a two-step process with an equilibration/pre-acidification reactor followed by a methane reactor. Cell suspensions from the microbial community in the reactor generated similar amounts of ammonia and formic acid from cyanide, with little formamide accumulation. Optimal cyanide removal took place at pH 6–7.5 and temperature 25–37 °C (Siller and Winter 1998b). Sludge from an anaerobic lagoon has been also used in an UASB reactor for successful treatment of a cyanide-containing tapioca starch wastewater. High gas productivity and up to 98% cyanide removal was achieved for 25 m/L cyanide in the feed, requiring 15 days for the complete recovery of the reactor (Annachhatre and Amornkaew 2001).

Industrial wastewaters from steel manufacturing, fuel processing, coal conversion, and coking, which contain high concentrations of ammonia, phenol, thiocyanate, and cyanide, are also amenable to biodegradation by methanogenic consortia (Fedorak and Hrudey 1989). Thus, up to 98% cyanide was successfully removed during methanogenic degradation of phenol in an UASB reactor fed with 20 mg/L cyanide (Kumar et al. 2011). Sequential anaerobic-aerobic bioreactors have been also used for the treatment of complex mixtures of phenol, ammonia, thiocyanate, and cyanide (Chakraborti and Veeramani 2006). In a combined anaerobic-aerobic system treating coking wastewater with hydraulic retention time of 114 h, 81.8% chemical oxygen demand (COD), 85.6% total organic carbon (TOC), 99.9% total phenols, 98.2% thiocyanate, and 85.4% cyanide were removed (Joshi et al. 2016). Microbial diversity in both anaerobic and aerobic reactors was also analyzed resulting that phenol-degrading and hydrolytic bacteria such as *Ottowia*, *Soehngenia*, and *Corynebacterium* were predominant in the anaerobic sludge, whereas thiocyanate and phenol degraders belonging to *Thiobacillus*, *Diaphorobacter*, and *Comamonas* genera were most abundant in the aerobic sludge. *Methanosarcina* was the dominant archaea in the anaerobic reactor (Joshi et al. 2016). Similar removal efficiencies were obtained in a previous study using a combination of anaerobic-aerobic-anoxic bioreactors for the treatment of coke wastewater (Sharma and Philip 2015), highlighting the feasibility to apply successfully this sequential anaerobic-aerobic operation to treat complex phenol and cyanide-containing wastewaters.

The cyanidation process used for extraction of gold and other metals from ores in mining activities generates large amounts of cyanide-containing wastes that require treatment before they can be released to the environment (Luque-Almagro et al. 2016; Mekuto et al. 2016). Different approaches have been applied to remove cyanide from cyanidation and electroplating wastewater, but they operate basically under aerobic conditions (Akcil and Mudder 2003; Sirianuntapiboon et al. 2008; Kuyucak and Akcil 2013; Mekuto et al. 2016). However, laboratory and engineered wetland experiments based on aerobic and anaerobic processes have been used for the construction of a pilot field-scale passive system at a gold mine in northern Spain (Álvarez et al. 2013). In a laboratory test with two anaerobic columns operating in a continuous flow-through mode, one filled from bottom to the top with a 20-cm layer of limestone and a 100-cm layer of compost, and the other with the same content but including grained iron particles mixed with the compost substrate, the cyanidation wastewater was remediated with a 60–70% reduction of weak acid dissociable cyanides. Remediation of cyanide was slightly higher in the column with iron, probably due to the formation of complexes that could be adsorbed in the compost. In addition, the compost-based constructed wetlands detoxify the cyanidation effluents, successfully removing both weak acid dissociable cyanide and metals like copper (more than 90%). Therefore, aerobic/anaerobic wetland-based passive systems can be considered as a suitable technology for remediation of mining cyanidation effluents (Álvarez et al. 2013). However, the role of biodegradation in this system was not analyzed and probably the main mechanisms involved in cyanide removal were of physical/chemical nature, like complexation to solid phases and photodegradation.

Under anaerobic conditions, sulfate-reducing bacteria could be also used for an efficient biodegradation of cyanide and metal-containing wastewaters (Song et al. 1998; Quan et al. 2004). Thus, it has been demonstrated that removal of both free cyanide and metal-cyanide complexes (mainly with zinc, nickel, or copper) may occur under sulfate reduction conditions using a granular sludge from an UASB reactor fed with brewery wastewater and enriched with sludges from electroplating and industrial wastewater plants. Analysis of the microbial community revealed that a bacterial consortium composed of three major phylotypes including *Desulfovibrio* was responsible of cyanide degradation during sulfate reduction. In addition, sulfate-reducing bacteria were found to be less sensitive to cyanide than methanogenic bacteria (Quan et al. 2004). Therefore, sulfate reduction conditions may be used for a plausible treatment of industrial wastewaters containing cyanide and metal-cyanide complexes.

## Biodegradation of thiocyanate and nitriles

Anaerobic biodegradation of cyanide in the presence of sulfide can produce thiocyanate. In addition, most sulfide minerals have the potential to generate thiocyanate, a process accelerated in anaerobiosis and low alkaline conditions, usually generating cyanate, nitrate, and ammonium as breakdown products (Kuyucak and Akcil 2013). Thiocyanate can be also formed by the transfer of sulfur from thiosulfate to cyanide in the reaction catalyzed by the rhodanese. It has been described that an extracellular rhodanese from *Coprothermobacter* is involved in anaerobic cyanide metabolism (Tandishabo et al. 2007).

Bacteria able to degrade thiocyanate have been isolated from various aerobic and anaerobic environments like soils, soda lakes, gold mine tailing, and activated sludge. These organisms can use thiocyanate as an energy, carbon, sulfur, or nitrogen source (Sorokin et al. 2001; Gould et al. 2012; Watts and Moreau 2016). Most thiocyanate-degrading chemolithotrophic bacteria oxidize aerobically the sulfide released in thiocyanate degradation, but some species like *Thioalkalivibrio thiocyanodenitrificans* are facultative anaerobes capable of growth anaerobically with thiocyanate as electron donor and with nitrate or nitrite as electron acceptor (Sorokin et al. 2004). Heterotrophic bacteria are also capable of thiocyanate degradation, using this compound as a source of nitrogen (Watts and Moreau 2016). There are two mechanisms for thiocyanate degradation, the carbonyl sulfide and the cyanate pathways (Fig. 1), and both are essentially aerobic. Therefore, the different systems developed for thiocyanate biodegradation are usually aerobic, like the activated sludge tailing effluent remediation (ASTER™) process (Huddy et al. 2015; Kantor et al. 2015). Most of these bioremediation systems rely on microbial co-cultures or consortia that metabolize undesirable by-products or establish potential syntrophic links, increasing the robustness of the system (Gould et al. 2012; Watts and Moreau 2016). However, the development of novel bioreactor designs, such as the utilization of several aerobic/anaerobic/anoxic reactors in series, and the better understanding of the microbial populations and processes involved in the biodegradation processes, using modern techniques of global analysis that provides a holistic view, will allow the development of more efficient and effective bioremediation approaches.

A system with two trains (A and B) of four-stage moving bed biofilm reactors, with an anoxic operating reactor in the train B, was designed for thiocyanate, cyanate, and ammonia biodegradation of gold extraction wastewater (Villemur et al. 2015). These three contaminants were completely removed and *Thiobacillus* strains were identified as the predominant bacteria in all reactors, although the higher content of anammox-related bacteria in train B suggests that the nitrogen dissimilation process takes place by this route (Villemur et al. 2015). A set of two continuous culture reactors, the first maintained aerobic and the second operating anaerobically, was applied at the low temperature typical for boreal climate for using the thiocyanate and thiosulfate present in a gold extraction wastewater as denitrification electron donors. Interestingly, the anaerobic reactor showed a higher diversity of microbial genera than the aerobic-operating reactor (Broman et al. 2017).

Meta-omics (metagenomics and metaproteomics) applied to study the microbial communities degrading thiocyanate and cyanide in aerobic continuous-flow bioreactors revealed the dominance of *Thiobacillus* strains capable of thiocyanate degradation (Kantor et al. 2015; Rahman et al. 2016; Kantor et al. 2017). A large portion of bioreactor community was autotrophic, relying on the energy generated from oxidation of sulfur compounds produced during thiocyanate degradation. Genes involved in ammonium oxidation and denitrification, as required for complete nitrogen removal, were also detected (Kantor et al. 2015).

Nitrilases, enzymes that convert organic cyanides into their respective carboxylic acid and ammonia, have acquired a relevant position in industry because they have been applied in the synthesis of numerous compounds, and are considered an economic and environmental friendly alternative to chemical methods (Gong et al. 2012; Luque-Almagro et al. 2016; Park et al. 2017). Nitrilase substrates can be aliphatics, like glutaronitrile, or aromatics, like benzonitrile (Estepa et al. 2012). Most nitrilases works aerobically under mesophilic conditions, and therefore, microbial degradation of a wide range of nitriles, using free or immobilized cells, in batch or continuous-flow bioreactors, was investigated under aerobic conditions (Kobayashi and Shimizu 2000; Kao et al. 2006; Chen et al. 2010; Maniyan et al. 2013). Nevertheless, several nitrilases can also function anaerobically at elevated temperatures. Thus, a termostable nitrilase from the hyperthermophile *Pyrococcus* sp. M24D13, which was isolated under strict anaerobic conditions from soil samples from Antarctica, has been purified and characterized. This nitrilase showed optimal activity at 85 °C and pH 7.5 with benzonitrile and butyronitrile, its major substrates. The enzyme also showed cyanidase activity (Dennet and Blammey 2016).

## Conclusions and future perspectives

Industrial wastewaters containing cyanide and related compounds like nitriles and thiocyanate may be bioremediated under aerobic conditions, but very little information is available about anaerobic cyanide biodegradation. Reductive or hydrolytic pathways may operate under anaerobic conditions, although cyanide degradation occurs more slowly and less successfully in comparison with the aerobic biodegradation. Biogas production associated to anaerobic biological treatments of wastewaters is an attractive technology. Anaerobic environments are found in nature, and cyanide-containing spills may occur where anaerobiosis is established. Isolation of novel microorganisms able to tolerate and degrade cyanide from both aerobic and anaerobic zones, application of microbial co-cultures or consortia, and acclimatization of the microbial communities to cyanide are approaches that could improve the degradation rates of food industry, gold mining, and other industrial effluents. Development of novel bioreactor designs utilizing sequential aerobic/anaerobic/anoxic systems and application of metagenomics and metaproteomics global analysis techniques that provide a holistic view for a better understanding of the mechanisms involved in the biodegradation processes and the composition and dynamics of microbial populations will contribute to develop more efficient and effective removal technologies.
